# Whole-Genome Sequencing Has the Potential To Improve Treatment for Rifampicin-Resistant Tuberculosis in High-Burden Settings: a Retrospective Cohort Study

**DOI:** 10.1128/jcm.02362-21

**Published:** 2022-03-16

**Authors:** Helen Cox, Galo A. Goig, Zubeida Salaam-Dreyer, Anzaan Dippenaar, Anja Reuter, Erika Mohr-Holland, Johnny Daniels, Patrick G. T. Cudahy, Mark P. Nicol, Sonia Borrell, Miriam Reinhard, Anna Doetsch, Christian Beisel, Sebastien Gagneux, Robin M. Warren, Jennifer Furin

**Affiliations:** a Division of Medical Microbiology, Department of Pathology, University of Cape Town, Cape Town, South Africa; b Institute of Infectious Disease and Molecular Medicine and Wellcome Centre for Infectious Disease Research, University of Cape Town, Cape Town, South Africa; c Swiss Tropical and Public Health Institute, Basel, Switzerland; d University of Basel, Basel, Switzerland; e Tuberculosis Omics Research Consortium, Family Medicine and Population Health, Institute of Global Health, Faculty of Medicine and Health Sciences, University of Antwerp, Antwerp, Belgium; f Médecins Sans Frontières, Khayelitsha, Cape Town, South Africa; g Section of Infectious Diseases, Department of Internal Medicine, Yale School of Medicine, New Haven, Connecticut, USA; h Division of Infection and Immunity, School of Biomedical Sciences, University of Western Australia, Perth, Australia; i Department of Biosystems Science and Engineering, ETH Zürich, Basel, Switzerland; j DSI-NRF Centre of Excellence for Biomedical Tuberculosis Research/SAMRC Centre for Tuberculosis Research, Division of Molecular Biology and Human Genetics, Faculty of Medicine and Health Sciences, Stellenbosch University, Stellenbosch, South Africa; k Department of Global Health and Social Medicine, Harvard Medical School, Boston, Massachusetts, USA; University of Iowa College of Medicine

**Keywords:** tuberculosis, treatment, drug resistance, whole-genome sequencing, *Mycobacterium tuberculosis*

## Abstract

Treatment of multidrug-resistant or rifampicin-resistant tuberculosis (MDR/RR-TB), although improved in recent years with shorter, more tolerable regimens, remains largely standardized and based on limited drug susceptibility testing (DST). More individualized treatment with expanded DST access is likely to improve patient outcomes. To assess the potential of TB drug resistance prediction based on whole-genome sequencing (WGS) to provide more effective treatment regimens, we applied current South African treatment recommendations to a retrospective cohort of MDR/RR-TB patients from Khayelitsha, Cape Town. Routine DST and clinical data were used to retrospectively categorize patients into a recommended regimen, either a standardized short regimen or a longer individualized regimen. Potential regimen changes were then described with the addition of WGS-derived DST. WGS data were available for 1274 MDR/RR-TB patient treatment episodes across 2008 to 2017. Among 834 patients initially eligible for the shorter regimen, 385 (46%) may have benefited from reduced drug dosage or removing ineffective drugs when WGS data were considered. A further 187 (22%) patients may have benefited from more effective adjusted regimens. Among 440 patients initially eligible for a longer individualized regimen, 153 (35%) could have been switched to the short regimen. Overall, 305 (24%) patients had MDR/RR-TB with second-line TB drug resistance, where the availability of WGS-derived DST would have allowed more effective treatment individualization. These data suggest considerable benefits could accrue from routine access to WGS-derived resistance prediction. Advances in culture-free sequencing and expansion of the reference resistance mutation catalogue will increase the utility of WGS resistance prediction.

## INTRODUCTION

The last several years have seen radical changes in the way that persons living with multidrug-resistant or rifampicin-resistant tuberculosis (MDR/RR-TB) are treated for their disease (1). In the past, recommended therapeutic regimens lasted 18 to 24 months, included multiple medications with a high pill burden, and relied on toxic yet ineffective injectable agents, leading to a global success rate of just over 50% (2). More recently, however, the World Health Organization (WHO) has recommended shorter, all-oral regimens, based on newer and repurposed drugs such as bedaquiline, linezolid, and clofazimine, for a majority of patients newly diagnosed with MDR/RR-TB (3).

While the use of all-oral regimens lasting 9 to 12 months has the potential to revolutionize the treatment of MDR/RR-TB, few countries have moved to implement them on a programmatic level (4). Those that have done so tend to recommend a “standardized” all-oral shorter regimen for those meeting certain eligibility criteria, based on previous treatment history, and limited drug susceptibility testing (DST) results (5, 6). Those ineligible for the standardized short regimen often receive a longer, all-oral regimen. Although eligibility criteria differ, most programs exclude from shorter therapy individuals whose Mycobacterium tuberculosis strains have documented fluoroquinolone resistance or those whose strains may be at risk for fluoroquinolone resistance (including patients with previous exposure to second-line antituberculosis medications), documented resistance to injectable agents, extensive pulmonary disease, and factors that may predict more extensive second-line resistance, for example, the presence of both the *inhA* and *katG* mutations conferring isoniazid resistance in South Africa (based on unpublished surveillance data).

There are several challenges with this approach. First, factors associated with fluoroquinolone resistance do not correlate with actual resistance in many instances, which could lead to excluding patients who would benefit from shortened treatment regimens, as well as including patients who are unlikely to be successfully treated (7). Second, the standardized all-oral shorter regimen recommended by WHO includes multiple drugs that are unlikely to be effective for many patients with MDR/RR-TB; including isoniazid, pyrazinamide, ethambutol, and ethionamide, which significantly add to the pill burden and toxicity of treatment (3, 8). Finally, patients needing to receive a longer individualized regimen, which uses core backbone drugs with additional agents added as needed according to an evidence-based, WHO-specified rank order (3), often require a more comprehensive drug susceptibility profile to guide regimen construction. Limited DST in these circumstances could lead to inclusion of ineffective drugs and exclusion of effective drugs making treatment less effective, more toxic, and challenging to complete (9). Less effective treatment can also lead to the generation of additional resistance mutations and transmission of drug-resistant tuberculosis (10).

Whole-genome sequencing (WGS), where all known resistance-conferring mutations are identified simultaneously, is increasingly being used to provide comprehensive DST to individualize MDR/RR-TB treatment in well-resourced settings (11, 12). In support, WHO has released technical guidance on the use of next-generation sequencing to infer *M. tuberculosis* drug resistance (13) and, more recently, released a catalogue of mutations conferring *M. tuberculosis* drug resistance, which will be updated regularly as more data emerges (14). However, WGS-derived DST profiles do not necessarily need to be used to fully individualize treatment. In high-burden settings, concerns about the clinical expertise required to provide fully individualized regimens for MDR/RR-TB potentially limit implementation (13, 15). South Africa currently recommends a standardized treatment approach utilizing a shorter, all-oral regimen for MDR/RR-TB (6). In order to demonstrate how WGS-derived DST may be used to provide more appropriate and effective treatment within a semi-standardized treatment algorithm, we retrospectively assessed potential regimen changes indicated by WGS for a large cohort of MDR/RR-TB patients diagnosed in Khayelitsha, Cape Town, South Africa.

## MATERIALS AND METHODS

### Study design.

This analysis applied current treatment recommendations to a retrospective cohort of MDR/RR-TB patients diagnosed between 2008 and 2017 in Khayelitsha, a subdistrict of Cape Town. Khayelitsha has a population of approximately 450,000, with high burdens of HIV, TB, and MDR/RR-TB. A detailed prospective clinical database was established in Khayelitsha to evaluate a decentralized program to diagnose and treat RR-TB from late 2007 onward (16). Anonymized clinical data were linked to WGS data derived from stored *M. tuberculosis* isolates held in a biobank at Stellenbosch University. Ethical approval for collection of *M. tuberculosis* isolates in the biobank was granted by the Stellenbosch University Research Ethics Committee and approval for linking WGS data to clinical data were granted by the University of Cape Town Human Research Ethics Committee. All patients routinely diagnosed with MDR/RR-TB between 2008 and 2017 and with pretreatment (defined as up to 1 month after second-line treatment initiation) *M. tuberculosis* isolate WGS data available were included in the study cohort.

### Current South African MDR/RR-TB treatment guidance.

The standardized short (9 to 12 month) regimen for MDR/RR-TB in South Africa is composed of an intensive phase of 4 to 6 months duration containing; bedaquiline (minimum 6 months), linezolid (2 months), high-dose isoniazid, levofloxacin, clofazimine, pyrazinamide, and ethambutol. The 5-month continuation phase is composed of levofloxacin, clofazimine, pyrazinamide, and ethambutol. If patients’ *M. tuberculosis* strains develop resistance or if the patients develop intolerance to pyrazinamide, ethambutol, or isoniazid, these drugs can be removed from the regimen without replacement. If more than one drug is removed, either bedaquiline or linezolid treatment is lengthened.

Patients who do not qualify for a short regimen are offered a long 18- to 20-month regimen with a 6- to 8-month intensive phase containing five drugs, as well as a continuation phase containing four drugs. Exclusion criteria for the standardized short regimen include the following: prior exposure to >1-month second-line TB treatment; complicated extrapulmonary TB (EPTB); close contact with XDR-TB (based on the pre-2021 definition of MDR-TB with both fluoroquinolone and second-line injectable resistance) or pre-XDR (MDR-TB with either fluoroquinolone or second-line injectable resistance); age <6 years; extensive TB disease on chest X-ray; the presence of both *katG* and *inhA* isoniazid resistance-conferring mutations; and suspected or confirmed resistance to fluoroquinolones, injectable agents, bedaquiline, clofazimine, or linezolid. Patients fulfilling any one of these criteria receive an individualized long regimen (6). Drugs are chosen according to WHO guidance, starting with categories A and B; with drug choice dependent on available DST, contraindications, and the location of disease (6).

Currently, all individuals under investigation for pulmonary TB are tested with Xpert MTB/RIF Ultra (Xpert, Cepheid, Sunnyvale, CA), and culture if Xpert is negative for individuals living with HIV. If rifampicin-resistance is detected, a first-line line probe assay (LPA; Hain Lifescience MTBDR*plus*, Tübingen, Germany) is used for isoniazid DST. If isoniazid susceptibility is demonstrated by LPA, phenotypic INH DST is conducted on a cultured specimen (allowing for confirmation of rifampicin resistance). A second-line LPA (MTBDR*sl;* Hain Lifescience, Nehren, Germany) is used to detect resistance to fluoroquinolones and second-line injectables, with isolates that test susceptible subjected to phenotypic fluoroquinolone (levofloxacin) DST. Isolates with fluoroquinolone resistance are subjected to extended DST to a wider range of second-line drugs, including bedaquiline and linezolid. All DST conducted programmatically, both phenotypic and genotypic, is referred to as routine DST. Rifampicin monoresistant TB (RMR-TB) was defined as rifampicin resistance and isoniazid susceptibility.

### Whole-genome sequencing.

Stored *M. tuberculosis* isolates were recultured for DNA extraction. WGS was performed on libraries prepared from purified genomic DNA using Illumina Nextera XT library and NEBNext Ultra TM II FS DNA Library Prep kits. Sequencing was performed using the Illumina HiSeq 2500 or NextSeq 500 platforms. Raw FASTQ WGS data with a minimum coverage depth of 20× of the *M. tuberculosis* H37Rv reference genome, were analyzed using TBProfiler (command line, version 2.8.12) to determine *M. tuberculosis* drug resistance-conferring mutations (17).

### Data analysis.

Clinical and laboratory data pertaining to the exclusion criteria for the standardized short regimen were extracted from the routine database. Data were available for: previous second-line TB treatment, routinely diagnosed second-line drug resistance (fluoroquinolones and injectable agents), age, the presence of extrapulmonary disease, and the presence of both isoniazid resistance-conferring mutations (*katG* and *inhA*). Data were not available for situations where second-line resistance was suspected, extensive disease on X-ray, or complicated EPTB. While EPTB was recorded on the clinical database, the disease site was not consistently recorded. Therefore, all EPTB was considered complicated, with consequent exclusion from the standardized short regimen. Data on the presence of isoniazid resistance-conferring mutations, derived routinely from the LPA, were also not consistently available. Where routine mutation data were missing, mutation data based on WGS were used instead. These data, related to the exclusion criteria, were used to categorize patients as receiving either the currently recommended standardized short or longer individualized regimen.

Final DST profiles were derived from a combination of both routine and WGS-based DST results, where resistance on any test was categorized as likely resistance, i.e., in the case of discordance between routine and WGS-based DST, a conservative approach of assuming resistance was taken. Final DST profiles were then used to determine the appropriateness of initial categorization into the standard shorter regimen and the longer individualized regimen and to describe how treatment regimens might change based on the current South African guidance, were WGS-derived resistance profiles available to the treating clinician, in addition to routine DST.

### Data availability.

All sequencing data are available via online repository (European Nucleotide Archive) under accession number PRJEB45389. A limited deidentified data set containing patient-level data will also be made available on publication (https://datadryad.org/stash).

## RESULTS

### Cohort description.

WGS data from pretreatment *M. tuberculosis* isolate WGS data were available for 1,274 patient treatment episodes between 2008 and 2017 inclusive. The mean read depth at drug resistance-conferring sites was 82 (95% confidence interval = 79 to 85). There were no major differences between patients with WGS data available and those without, as previously described (18). Among these patient episodes, 897 (70.4%) were HIV positive, 593 (46.5%) were female, and second-line treatment was initiated in 1,162 (91.2%).

If this cohort were to be treated under current treatment recommendations, the most common reason for exclusion from the standardized short regimen would have been demonstrated resistance to a fluoroquinolone or a second-line injectable (Table 1). Overall, 834 (65.5%) patient episodes would have been eligible for the standardized short regimen.

**TABLE 1:** Presence of exclusion criteria for the standardized short regimen^*a*^

Exclusion criteria	*n* (%)
None	834 (65.5)
At least one exclusion criteria	440 (34.5)
Resistance to FLQ and/or INJ on routine DST	242 (19.0)
Both INH resistance-conferring mutations (*katG* and *inhA*)	196 (15.4)
EPTB	113 (8.9)
Previous second-line TB treatment > 1 mo	60 (4.7)
Age < 6 yrs	7 (0.5)

a Note that individual patient episodes may have more than one exclusion criteria. FLQ, fluoroquinolone; INJ, second-line injectable; DST, drug susceptibility testing; EPTB, extrapulmonary TB.

### Regimen changes based on merged routine and WGS-derived resistance profiles.

There was considerable discordance between DST profiles based on WGS and those from routine diagnosis (Table 2). Overall, 104 (8.2%) isolates had no rifampicin resistance detected on WGS. Notably, fluoroquinolone resistance was detected in an additional 24 isolates using WGS. Based on final (merged routine and WGS-derived DST) profiles, 254 (19.9%) patients were classified as having RMR-TB, 822 (64.5%) had MDR-TB without fluoroquinolone resistance, and 198 (15.5%) had MDR-TB with fluoroquinolone resistance (Table 3).

**TABLE 2:** Differences between routine MDR/RR-TB diagnostic classification and WGS-derived resistance profiles^*a*^

WGS-derived resistance profile	Routine drug resistance profile (no. of samples)						Total
	RR-TB^*b*^	RMR-TB	MDR-TB^*c*^	PreXDR-FLQ	PreXDR INJ	XDR-TB	
RS-TB	5	32	63	1	2	1	104
RMR-TB		225	12	0	0	1	238
MDR-TB		9	654	6	11	1	681
PreXDR-FLQ			23	64	1	10	98
PreXDR INJ		1	11	0	48	3	63
XDR-TB			2	14	10	64	90
Total	5	267	765	85	72	80	1,274

a RS-TB, rifampicin-susceptible TB; RMR-TB, rifampicin monoresistant TB (defined as rifampicin resistance and isoniazid susceptibility); PreXDR FLQ, MDR-TB plus fluoroquinolone resistance; PreXDR INJ, MDR-TB plus second-line injectable resistance; XDR-TB, extensively drug-resistant TB (defined as MDR-TB plus both fluoroquinolone and second-line injectable resistance).

b Diagnosed on Xpert MTB/RIF only (no further DST).

c Routine second-line DST not available for all MDR-TB patient episodes.

**TABLE 3:** Final drug resistance profiles, based on merged routine and WGS data^*a*^

Drug resistance category	Profiles	*n*
RMR-TB (*n* = 254)		
	R	244
	R, ETO	4
	R, INJ	3
	R, FLQ	1
	RE	1
	RE, ETO	1
MDR-TB (FLQ susceptible) (*n* = 822)		
	RH	137
	RH, ETO	122
	RHZES, ETO	110
	RHZE, ETO	66
	RHZS, ETO	61
	RHE, ETO	56
	RHZES, INJ, ETO	46
	RHES	37
	RHZES	31
	RHE	29
	RHS, ETO	29
	RHES, ETO	14
	RHZE	14
	RHS	9
	RHZES, INJ, ETO, CYC	8
	RHZS	8
	RHZS, INJ, ETO	7
	RHZ	6
	RHS, INJ, ETO	5
	RHZES, ETO, CYC	4
	RHZS, PAS	4
	RHE, INJ, ETO	3
	RZ	3
	RH, INJ	2
	RHZ, ETO	2
	RHZE, INJ	2
	RHES, INJ	1
	RHZ, FLQ, INJ, ETO	1
	RHZE, ETO, CYC	1
	RHZE, INJ, ETO	1
	RHZES, PAS	1
	RZES	1
	RZS, ETO	1
MDR-TB (FLQ resistant) (*n* = 198)		
	RHZES, FLQ, INJ, ETO	57
	RHZES, FLQ, ETO	36
	RHZE, FLQ, ETO	24
	RHZES, FLQ, INJ, ETO, CYC	14
	RHE, FLQ, ETO	9
	RHZS, FLQ, ETO	7
	RHES, FLQ, INJ, ETO	6
	RHZE, FLQ, INJ, ETO	5
	RHZE, FLQ, INJ, ETO, CYC	5
	RHZES, FLQ, INJ	5
	RHZES, FLQ, INJ, ETO	5
	RHZE, FLQ, ETO, CYC	4
	RHE, FLQ, INJ, ETO	3
	RH, FLQ, ETO	2
	RHE, FLQ	2
	RHES, FLQ	2
	RHZE, FLQ, INJ, ETO	2
	RHZES, FLQ, ETO, CYC	2
	RHZES, FLQ, ETO, PAS	2
	RH, FLQ	1
	RH, FLQ, INJ	1
	RHES, FLQ, ETO	1
	RHZE, FLQ	1
	RHZES, FLQ	1
	RHZES, FLQ, INJ, ETO, PAS	1

a R, rifampicin; H, isoniazid; Z, pyrazinamide; E, ethambutol; S, streptomycin; FLQ, fluoroquinolone; INJ, second-line injectable; ETO, ethionamide; CYC, cycloserine; PAS, *para*-aminosalicylic acid.

**FIG 1:**
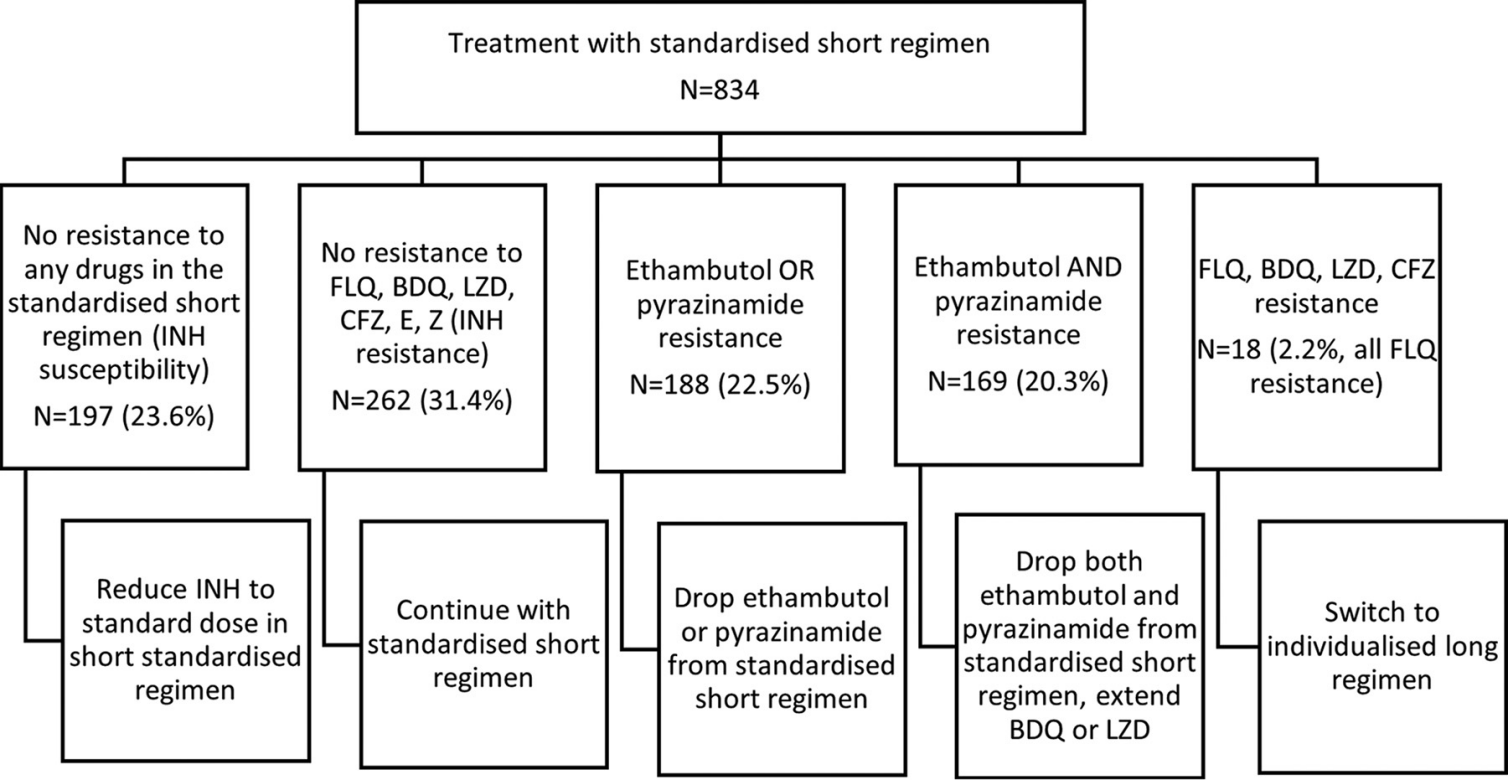
Changes to treatment based on drug resistance profile (combined routine and WGS DST data) for patients who would have been started on the standardized short regimen.

Figure 1 indicates potential regimen changes for patients who would have been treated with the standardized short regimen (under current treatment guidelines) given the additional DST data provided by WGS. Overall, 197 (23.6%) patients had RMR-TB (isoniazid susceptible RR-TB) and could potentially have received the standard isoniazid dose. A further 262 (31.4%) would not have required any change to the short, standardized regimen. However, 188 (22.5%) patients had evidence of either ethambutol or pyrazinamide resistance and could potentially have had these drugs removed from the standardized regimen. The standardized short regimen may have been compromised for the 169 (20.3%) patients with both ethambutol and pyrazinamide resistance; these patients would have benefited from changes to the regimen as per national guidance. Finally, 18 (2.2%) patients would have needed to be switched to a longer individualized regimen, due to additional fluoroquinolone resistance (not detected routinely).

**FIG 2:**
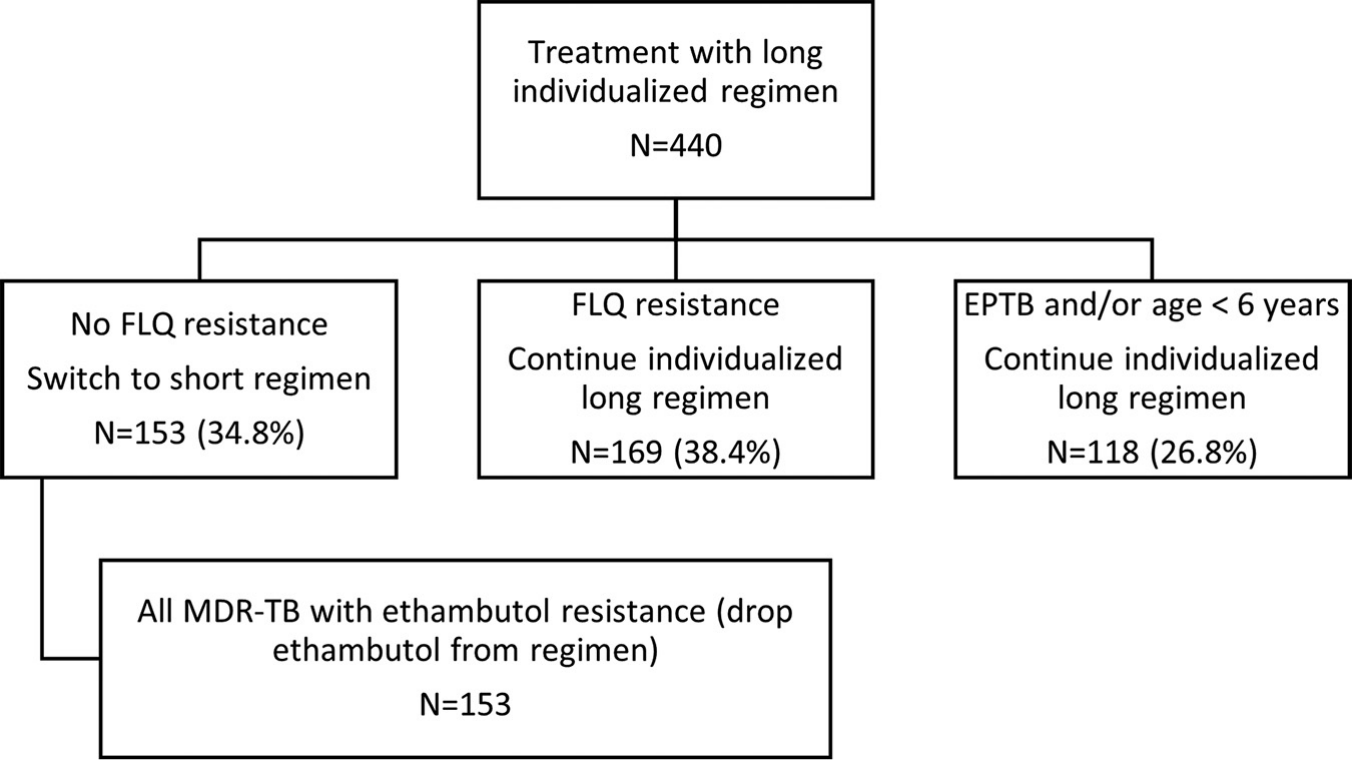
Changes to treatment based on drug resistance profile (combined routine and WGS DST data) for patients who would have been treated with a longer individualized regimen.

Among the 440 patients who would not have been eligible for the short regimen, 153 (34.8%) could potentially have received the shorter regimen (Fig. 2). These are patients who did not have EPTB, were aged above 6 years and did not have resistance to the fluoroquinolones, bedaquiline, linezolid, or clofazimine (the key agents in the short regimen). In addition, all 153 patients could potentially have had ethambutol removed from the short regimen due to demonstrated resistance. Among the 322 patients who would have been excluded from the short regimen based on presence of both isoniazid resistance-conferring mutations or previous second-line TB treatment, only 169 (52.5%) actually had MDR/RR-TB with fluoroquinolone resistance.

## DISCUSSION

These data demonstrate that a significant proportion of MDR/RR-TB patients may benefit from more appropriate treatment regimens with access to expanded DST through WGS, even in a setting with relatively high access to routine line probe assay and phenotypic DST. Among patients who would have been eligible for the short regimen, 46% may have had their regimen adjusted through reducing dosage or removing ineffective drugs. A further 22% may have benefited from regimens that were adjusted to provide more effective treatment. Importantly, a third of patients not eligible for the short regimen could potentially have been re-allocated to the shorter regimen, with substantial patient benefits. These data also demonstrate that more complete DST data could be incorporated into an algorithmic approach to regimen allocation, such as that currently followed in South Africa, without adding significant complexity for clinicians in high MDR/RR-TB burden settings. They also show the potential importance of WGS data for providing information on resistance to medications such as ethambutol and pyrazinamide that are not commonly assessed in high-burden settings such as South Africa.

Overall, just over a fifth of all patients may be considered to have more complicated MDR/RR-TB requiring more individualized treatment. This includes young children, patients with EPTB and those with M. tuberculosis strains that have fluoroquinolone resistance. Fluoroquinolone resistance is strongly associated with poor treatment outcomes (19), and remains a key drug for MDR/RR-TB treatment (3). For these patients, the addition of detailed DST through WGS could enable the construction of more individualized regimens, as is the norm in low-burden, high-resource settings (20).

There are a number of challenges for the implementation of WGS for patient treatment in high-burden settings (15, 21). These include cost, laboratory infrastructure and appropriate bioinformatics skills. These challenges may be feasible to overcome as demonstrated by recent experience in the Kyrgyz Republic (22). Accurate prediction of drug resistance from genomic data is also a challenge across all settings. The latter is increasingly being addressed through curation of large, globally derived data sets describing associations between mutations and phenotypic resistance that have informed the recent WHO catalogue of resistance-conferring mutations (14). There are, however, gaps in this catalogue, particularly for new and repurposed drugs such as bedaquiline, delamanid, and linezolid. In this current data set, no mutations known to confer resistance to these drugs were identified with TBProfiler. This was likely due to the small number of included patients with prior use of these drugs, with most of these returning to treatment after prior loss to follow-up. However, as use of these drugs expands, it is likely that both resistance to these drugs and knowledge of genomic predictors of resistance will also increase. Drug resistance prediction from sequencing data is also becoming increasingly accessible with newer tools (23).

MDR/RR-TB treatment, while much improved, remains lengthy and difficult to adhere to for patients with concomitant poor overall patient outcomes (1, 24). Globally, only 57% of patients who started treatment in 2017 were successfully treated and at least 16% of patients did not complete the full treatment course. Reducing pill burden and removing ineffective, and therefore unnecessary, drugs that are often associated with adverse events is therefore likely to improve regimen tolerability and therefore treatment completion (25). In addition, increasing the proportion of patients who can be treated with shorter, more tolerable regimens is likely to have significant benefits. Individuals with rifampicin-resistant but isoniazid-susceptible disease (RMR-TB) are a particular patient group that may benefit significantly from more detailed DST. We have previously demonstrated, using this data set, that resistance to TB drugs other than rifampicin and isoniazid differs considerably between isolates categorized as RMR-TB and MDR-TB (18). The data presented here confirm that patients presenting with RMR-TB have RR-TB with very little resistance to other drugs. Given that RMR-TB composed approximately 20% of all patient episodes in this setting and 22% of MDR/RR-TB globally (24), there may be significant potential to simplify or shorten treatment for this group.

Currently, WGS is predominantly performed on DNA isolated from cultured *M. tuberculosis* isolates and is therefore subject to the same delays as for *M. tuberculosis* culture as a diagnostic tool. However, experience has demonstrated that replacing a series of molecular diagnostic tests with WGS resulted in shorter turnaround times (11). Encouragingly, there is now evidence that culture-free sequencing directly from specimens may be feasible (at least for sputum smear-positive patients) and could result in rapid actionable results (26–28). Indeed, culture-free sequencing could provide complete DST profiles upfront, allowing for the initiation of more appropriate treatment regimens from the start and reducing the risks of multiple changes to regimens as more DST results become available.

Among the limitations of this study was the considerable discordance in rifampicin susceptibility detected by WGS and that from routine DST, with 9% of sequenced isolates predicting rifampicin susceptibility. There are several potential explanations for this, including the capacity of sequencing to detect heteroresistance which is highly dependent on the depth of coverage obtained and the cutoff percentages of resistance-conferring mutations used to predict resistance. In addition, mixed strain infections, where drug-susceptible strains outgrow more resistant strains in culture, may result in discordance (29). Discordant DST results are relatively common and can be difficult for clinicians to interpret (30). In this analysis, we employed a conservative approach where any resistance, either detected through routine diagnostic testing or predicted through WGS, was classified as resistance. While such an approach can simplify DST interpretation, discordant rifampicin DST on routine testing could prompt additional clinical assessment and is an area where additional research into causative factors is warranted. A further potential limitation is the reliance on Xpert MTB/RIF for RR-TB diagnosis; while Ultra is more sensitive than its predecessor, both tests are less sensitive than TB culture performed for all individuals with presumptive TB. Finally, use of the more recent updated catalogue of resistance-conferring mutations may have reduced discordance and potentially identified resistance to some of the newer or repurposed drugs.

We considered some of the key factors used to allocate treatment regimens based on the South African guidance. However, there are several other factors considered by clinicians in deciding treatment regimens, including drug intolerance, heightened risk of adverse events associated with particular drugs, extent of disease, HIV treatment regimens, and patient preference. In addition, we did not consider pretreatment laboratory tests such as those for anemia and hepatic or renal function. Low hemoglobin levels are a relatively common exclusion criteria for the short regimen since these patients are likely not to tolerate linezolid. However, more comprehensive DST may enable patients with moderate or severe anemia to continue a short regimen that excludes linezolid. Our analysis was therefore not intended to provide definitive treatment regimens for individual patients. In addition, there are multiple shorter regimens, other than that used in South Africa, being considered for routine use or use under operational research conditions that do not contain isoniazid, pyrazinamide, and/or ethambutol, including the Nix-TB regimen, the ZeNIX-TB regimen, the endTB regimens, the PRACTECAL regimen, and the NEXT TB regimen (31). As a result, we may have overestimated the potential clinical benefit of WGS. It is important to note, however, that almost all the all-oral shorter regimens being assessed for MDR/RR-TB rely on fluoroquinolone resistance as a key inclusion/exclusion criterion and that identification of preexisting resistance to included drugs will become even more relevant as use (and therefore resistance) increases. Our findings therefore point to the continued potential benefits of WGS as newer treatment regimens are introduced.

Despite these limitations, these data from a very large patient cohort suggest that a significant proportion of patients would benefit from access to more detailed DST through WGS. While the observed discordance may preclude WGS completely replacing routine DST at this point, targeted sequencing may enable culture-free testing direct from specimens, providing more timely and accurate drug resistance prediction. In this study setting, many MDR/RR-TB patients are treated in primary care; this raises questions as to what types of support they might need to optimize the information received through WGS. These implementation questions can be addressed in further research. South Africa has already invested in WGS for management of MDR/RR-TB, which is currently performed at the National Health Laboratory Services National TB Laboratory upon specimen referral. For scale-up to occur, capacity is required at a provincial level and a standardized method for reporting results is needed.
